# Fine-Mapping the Branching Habit Trait in Cultivated Peanut by Combining Bulked Segregant Analysis and High-Throughput Sequencing

**DOI:** 10.3389/fpls.2017.00467

**Published:** 2017-04-04

**Authors:** Galya Kayam, Yael Brand, Adi Faigenboim-Doron, Abhinandan Patil, Ilan Hedvat, Ran Hovav

**Affiliations:** Department of Field Crops, Plant Science Institute, Agricultural Research OrganizationBet-Dagan, Israel

**Keywords:** peanut, branching habit, bulked segregant analysis, fine mapping

## Abstract

The growth habit of lateral shoots (also termed “branching habit”) is an important descriptive and agronomic character of peanut. Yet, both the inheritance of branching habit and the genetic mechanism that controls it in this crop remain unclear. In addition, the low degree of polymorphism among cultivated peanut varieties hinders fine-mapping of this and other traits in non-homozygous genetic structures. Here, we combined high-throughput sequencing with a well-defined genetic system to study these issues in peanut. Initially, segregating F_2_ populations derived from a reciprocal cross between very closely related Virginia-type peanut cultivars with spreading and bunch growth habits were examined. The spreading/bunch trait was shown to be controlled by a single gene with no cytoplasmic effect. That gene was named *Bunch1* and was significantly correlated with pod yield per plant, time to maturation and the ratio of “dead-end” pods. Subsequently, bulked segregant analysis was performed on 52 completely bunch, and 47 completely spreading F_3_ families. In order to facilitate the process of SNP detection and candidate-gene analysis, the transcriptome was used instead of genomic DNA. Young leaves were sampled and bulked. Reads from Illumina sequencing were aligned against the peanut reference transcriptome and the diploid genomes. Inter-varietal SNPs were detected, scored and quality-filtered. Thirty-four candidate SNPs were found to have a bulk frequency ratio value >10 and 6 of those SNPs were found to be located in the genomic region of linkage group B5. Three best hits from that over-represented region were further analyzed in the segregating population. The trait locus was found to be located in a ~1.1 Mbp segment between markers M875 (B5:145,553,897; 1.9 cM) and M255 (B5:146,649,943; 2.25 cM). The method was validated using a population of recombinant inbreed lines of the same cross and a new DNA SNP-array. This study demonstrates the relatively straight-forward utilization of bulk segregant analysis for trait fine-mapping in the low polymeric and heterozygous germplasm of cultivated peanut and provides a baseline for candidate gene discovery and map-based cloning of *Bunch1*.

## Introduction

Peanut (*Arachis hypogaea* L.) is an economically significant crop grown throughout the world. It is the second-most important cultivated grain legume and the fourth largest edible oilseed crop (Faostat, 1998). It is an unusual legume plant in that its flowers are borne aboveground, but the fruits develop underground. The plant is an indeterminate, annual herbaceous bush that is 15–70 cm tall and is comprised of an erect main shoot and a number of lateral shoots (branches) that begin at the base of the plant.

The growth angle of the lateral shoots (commonly referred to as “growth habit” or “branching habit”) is one of the most important descriptive characteristics of peanut (Pittman, 1995). The wild polyploid peanut species *A. monticola* usually has a spreading phenotype, in which lateral branches strike or partially strike the ground. In domesticated peanut, four different, and easily distinguishable categories of branching habit are known: prostrate, spreading, bunch, and erect (Table 1). The description of a new peanut variety, especially one that is of the Virginia marketing-type, will almost always begin with the definition of its growth habit (e.g., bunch or spreading). In addition to assisting breeders and other researchers in identifying accessions for specific traits, branching habit has a great impact on peanut physiology, productivity, and crop management. Since fruiting in peanut occurs underground, the distance between the flowering buds and the ground is an important factor. Pegs of bunch/erect plants that do not reach the ground will not produce pods on time. However, the pods of bunch/erect plants develop at the same time, promoting early maturation. The growth habit of peanut affects the implementation of agrotechnology such as mechanical cultivation and disease management (Butzler et al., 1998).

**Table 1:** **The four main types of growth habit in peanut (from Pittman, 1995)**.

**Phenotype**	**Illustration**	**Description**	**Example**
Prostrate	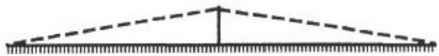	Branches that strike the ground and a conspicuous main stem	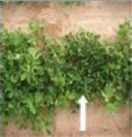
Spreading	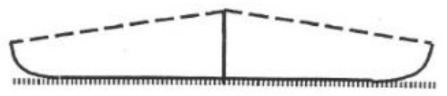	Branches partially on the ground, with their tips curved upward	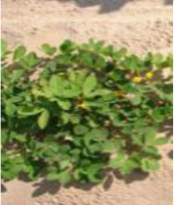
Bunch	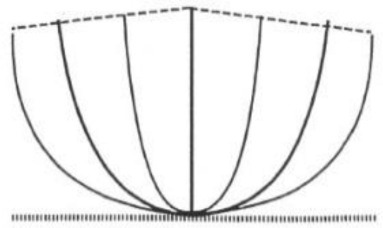	Semi-erect, branches curved upward, beginning at the base; main stem slightly taller than the others	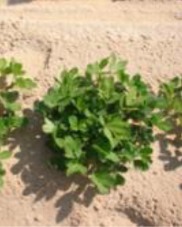
Erect	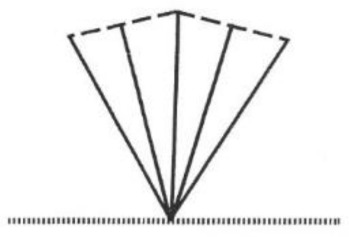	Branches grow straight up from the base and are generally 45° or less from vertical (*TAC1* style)	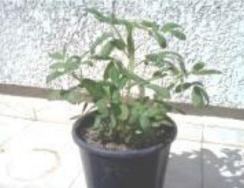

*The two growth habits represented in this study are marked in red*.

Despite the agronomic importance of the growth habit of peanut, both the inheritance of this trait and the genetic mechanism that controls branching habit in this crop are not clear. The trait was studied in detail during the 1960s and 1970s and, during that period, two distinct phenotypic groups were usually considered: the runner growth habit wherein the side branches are prostrate, always growing peripherally from the main axis, trailing on the ground except for the tips, which may be somewhat ascending and the bunch growth habit, in which the laterals are also erect or ascending. Initially, a two-gene model for the control of growth habit was suggested, with the runner habit dominant to the erect habit (Hull, 1933; Patel et al., 1936; Coffelt, 1974), but investigators had difficulty classifying the intermediate and/or abnormal growth habits of F_2_ progeny of crosses between plants exhibiting different growth habits. A fundamental set of experiments conducted by Ashri (1964, 1968) indicated the existence of a genic-cytoplasmic interaction that controls growth habit in peanut. In a project involving a series of reciprocal crosses, differences in growth habit were recorded. A few more nuclear and cytoplasmic genes were later identified by the same group and those researchers concluded that cytoplasmic inheritance has a major effect on the branching habit of peanut (Ashri, 1975; Ashri and Levi, 1975). In more recent studies, the branching habit trait was genetically characterized and mapped by using inter-specific crossing system with an amphidiploid species (Fonceka et al., 2012a,b). The branching habit trait was phenotyped quantitatively by using a continuous scale from 1 (procumbent) to 6 (erect). The trait showed a wide range of morphologies, ranging from completely prostrate to totally erect, and several QTLs were found to control the trait, with the most significant located on linkage groups a07, b05, a10, and b10.

Like many polyploid species, cultivated peanut has experienced a genetic bottleneck, which, together with the effects of domestication, has greatly narrowed its genetic diversity and limited DNA polymorphism among subsequently derived *Arachis* lines (Kochert et al., 1991; Moretzsohn et al., 2013). As a result, peanut has a low degree of polymorphism among cultivated varieties. This limited polymorphism has hindered the development of molecular and genomic tools for use in domesticated peanut. With the introduction of the genome sequences of peanut ancestors *Arachis duranensis* and *Arachis ipaensis* (Bertioli et al., 2016), peanut is now “the orphan legume genome whose time has come” (Ozias-Akins, 2013). These genome sequences provide the resources necessary to move peanut genomics to the next level, facilitating the development of SNP-based marker technologies. In the past, the most widely used molecular markers were simple-sequence repeats (SSRs). Despite their widespread use on the intra-species cultivated level (e.g., Selvaraj et al., 2009), the utility of SSR studies of peanut is limited by their apparent low frequency of across the genome and the relatively low-throughput method of analysis. The use of high-throughput markers like SNPs is necessary for efficient use of genomic data for marker-assisted selection, quantitative trait locus mapping and genomic selection. Recently, several platforms have been developed to facilitate the use of SNP markers for gene-mapping in peanut, including genotyping by sequencing (Zhou et al., 2014) and genome resequencing-based SNP arrays (Clevenger et al., 2016; Pandey et al., 2017). However, despite these advances, these platforms are usually highly efficient only for homozygous populations, like recombinant inbreed lines (RILs) and introgression lines (ILs), which are relatively tedious and expensive to construct in peanut. These methods are usually less effective for trait-mapping in heterozygous genetic populations (e.g., F_2_ and F_3_ generations) due to the allopolyploid nature of the peanut genome. This is particularly true in cases of genetic populations that are based upon a cross between closely related parental genotypes, which are occasionally needed for better genetic dissection of specific traits.

In this study, we used a well-defined genetic system to further investigate the genetic nature of the branching habit trait of peanut. Initially, segregating F_2_ populations derived from a reciprocal cross between very closely-related Virginia marketing-type cultivars were analyzed. Against this particular background, the spreading/bunch trait, a well-known characteristic of the Virginia varieties, was found to be controlled by a single gene with no cytoplasmic effect. Subsequently, a combination of bulked segregant analysis and deep sequencing was developed to facilitate the SNP detection process and the fine-mapping of this gene. The processes were validated using a RIL population derived from the same cross and a new SNP array (Pandey et al., 2017). The relatively straight-forward utilization of this technique in ultra-low polymorphic and highly heterozygous peanut germplasm is demonstrated.

## Materials and methods

### Plant material and data collection

Segregating F_2_ and F_3_ populations derived from a reciprocal cross between very closely-related Virginia-type peanut cultivars were studied. The parental lines were cv. “Hanoch,” a late-maturing spreading-type cultivar, and cv. “Harari,” a medium-maturing bunch-type cultivar. The two parental lines share substantial genetic background, since cv. Harari was developed from an initial cross between cv. Hanoch and cv. Shulamit and an additional back-cross of cv. Hanoch with cv. Hillah (the outcome of Hanoch × Shulamit). In 2013, 314, and 252 F_2_ individuals from reciprocal Hanoch × Harari and Harari × Hanoch crosses, respectively, were grown under field conditions. The plot consisted of two rows, 75 cm apart, with 40 cm spacing between plants within each row. The experimental plots were sown alongside commercial plots under full irrigation. All agricultural practices were carried out according to local growing protocols as described previously (Gupta et al., 2014).

Growth habit was recorded at 80 days after sowing. At the end of the season, pods were harvested on an individual-plant basis. For each sample, the total pod yield, net pod yield (where immature and unhealthy pods are excluded), number of pods, total seed weight, “dead-end” ratio (relative number of pods with the remote seed aborted) and seed ratio (net seed weight per plant divided by the net pod weight per plant) traits were recorded as well. From each population, approximately 150 F_3_ families were grown in the subsequent season, with 16 seeds from each F_2_ individual sown. Branching habit was recorded at the family level (spreading/bunch/segregating) at 80 days after sowing. Plant maturity index was determined based on three random plants in the homozygous F_3_ families, as the number of fully matured pods out of the total number of pods at 140 days after sowing. To validate the bulk segregant analysis, 94 RILs (F6:F8), which originated from the same Hanoch × Harari cross by single seed descent, were analyzed. In 2016, 16 randomly-selected plants from each RIL were grown under field conditions and the growth habit of each plant was recorded 70 days after sowing.

### RNA isolation, preparation of libraries, and high-throughput sequencing

Bulked segregant analysis was performed on the F_3_ families that were found to be homozygous for the spreading or bunch growth habit. In total, 52 completely bunch and 47 completely spreading families were sampled. Young leaves were collected from all 16 individuals in each family. In each phenotypic group (spreading/bunch), all tissues from the families were bulked for the RNA extraction. Working on the RNA level was preferable to working on the DNA genomic level due to the large and relatively complex peanut genome and also facilitated the detection of candidate genes. Samples were taken of each of the ground tissues (400 mg each) and were used for RNA extraction using the hot-borate method, as described by Brand and Hovav (2010). The total RNA was used to prepare two RNA-Seq libraries, using TruSeq RNA Sample Preparation Kit v2 (Illumina) following the manufacturer's protocol as described previously (Gupta et al., 2016). Libraries were validated using DNA Screen Tape D1000 and the Tapestation 2200 (Agilent). RNA-Seq libraries were sequenced using an Illumina HiSeqTM2000 (single lane) at the sequencing center at the Technion in Haifa, Israel.

Data analyses followed the general guidelines for bulk segregant analysis using next-generation sequencing (Magwene et al., 2011) and the specific guidelines for polyploids (Trick et al., 2012), with several modifications. Raw reads were subjected to a cleaning procedure using the FASTX Toolkit (http://hannonlab.cshl.edu/fastx_toolkit/ index.htm) including: (1) trimming read-end nucleotides with quality scores <30 using fastq_quality_trimmer and (2) removing reads with less than 70% base pairs with quality score ≤ 30 using fastq_quality_filter. The sequences were mapped against the 4X tetraploid peanut transcript assembly reference (http://www.peanutbase.org/) and against two *Arachis* diploid genomes (*A. duranensis* and *A. ipaensis*; Bertioli et al., 2016; peanutbase.org) using Bowtie2 aligner (Langmead and Salzberg, 2012). The genome Analysis Toolkit (GATK) Unified Genotyper software version 2.5.2 (McKenna et al., 2010; DePristo et al., 2011) was used for the detection of SNPs. A custom Perl script was used to derive the symmetric difference of the two SNP sets. Polymorphisms between homologous genomes generate the same doubled code and should be common to both SNP sets. Yet, differences in the SNPs between cv. Hanoch and cv. Harari (varietal-specific SNPs) should generate doubled code for only one bulk and, therefore, be unique to the corresponding SNP set. In this manner, ~13,000 varietal-specific SNPs were retrieved between the two bulks. These SNPs were further filtered according to the number of reads for each SNP > 50, GATK quality value >100 and BFR >3. Also, genes with SNP densities higher than 5 SNPs/kb were eliminated to avoid possible paralogue SNPs.

### Validation of the SNP markers and the bulk segregant analysis

For further validation of the SNPs, DNA was collected from the parental lines and 20 F_3_ progeny of the cross cv. Hanoch × cv. Harari using a DNA Easy kit (SIGMA Aldrich). The same leaves that were used for the RNA study were used for DNA extraction, but the DNA analysis was conducted on a single-plant basis instead of with bulk samples. To validate the three best SNP markers, the following primers were used: M35: F-TCTCTCTCTCTCACAGTCAC; R-CTTGCCGGCAAATAGAGCAT. M255: F--CAGATATGCAAGGCCTAACT; R-TGCCAGAGCAAGGAACATGT. M875: F-CCATCTGCAGTGAGAGTCAA; R-GTGATTCCTGCGTTCAAGTC. These primers were also used for further mapping of the trait in 182 F_4_ individuals derived from one F_3_ family segregating for the branching habit trait.

The fine-mapping of the branching habit gene carried out using the bulk-segregant approach was further validated by a custom Affymetrix Axiom SNP array (Pandey et al., 2017). For that analysis, DNA was collected from the two parental lines and each of 94 Recombinant Inbreed Lines (RILs) derived from the same cv. Hanoch × cv. Harari cross. Young leaves were collected from 12 random plants from each RIL and DNA was extracted with a specific kit (GenElute™; Sigma). DNA was quantified by Qubit (Invitrogene LTD) and diluted to 30 ng/uL according to the Affymetrix guidelines (http://www.affymetrix.com). The chip array calls were subjected to cluster-quality filtering, carried out according to Affymetrix guidelines, and additional filtering to select only SNPs that were polymorphic between the parental lines and segregated in a 1:1 ratio in the RILs.

## Results

### Branching habit is controlled by a single gene

**Figure 1:**
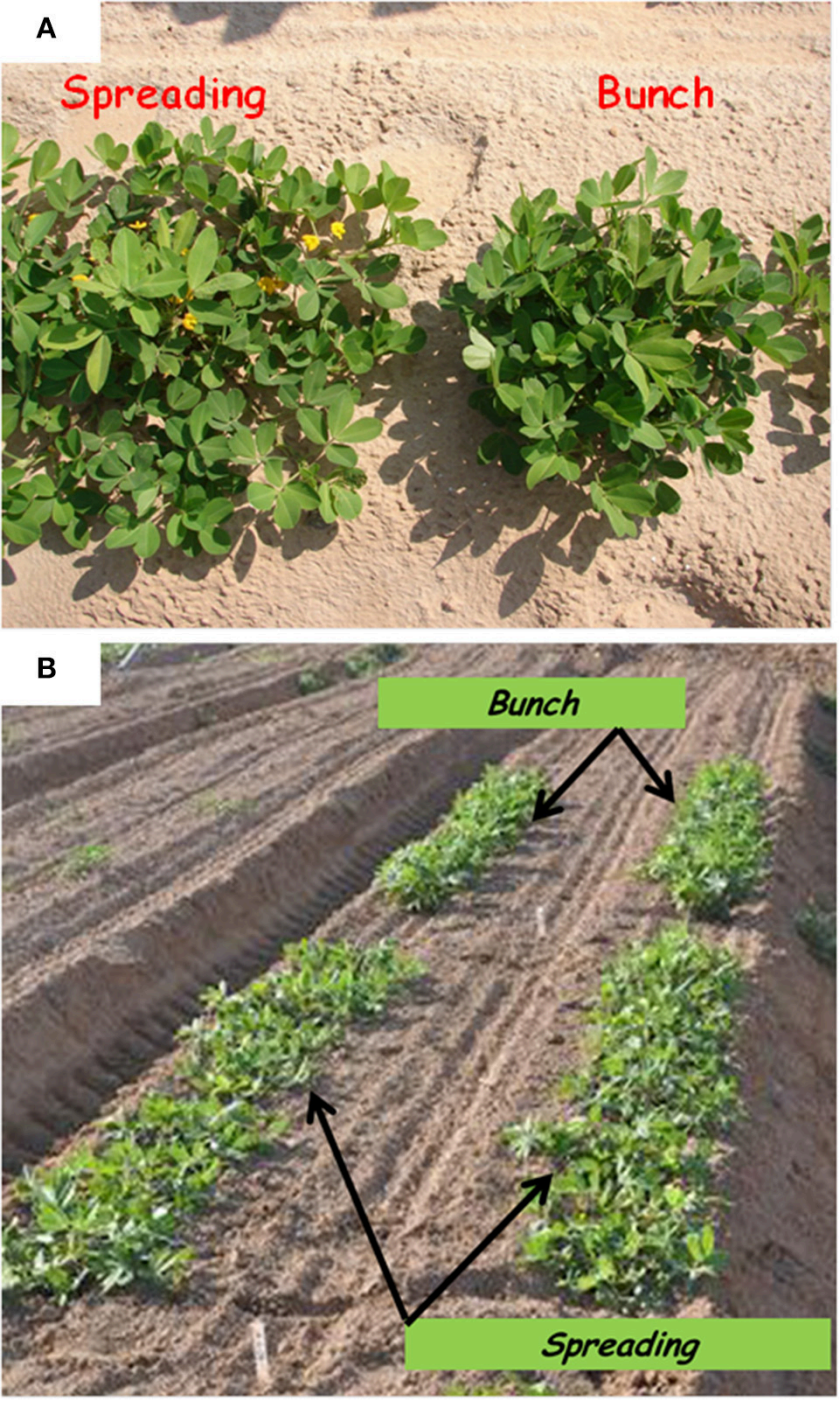
**Bunch and spreading phenotypes among (A)** F_2_ individuals and **(B)** F_3_ families grown under field conditions, t 50 days after sowing.

The segregation patterns of the branching habit trait in the F_1_, F_2_, and F_3_ generations of the “Hanoch” X “Harari” cross are presented in Table 2. As shown, in this genetic background, the spreading/bunch pattern appears to correspond to a single-gene model of inheritance with no cytoplasmic effect. The allele that confers the spreading phenotype is dominant over the one that confers the bunch habit, as demonstrated by the spreading phenotype of all the F_1_ hybrids, the 3:1 segregation ratio among the F_2_ progeny and the 1:2:1 segregation ratio among the F_3_ families. The gene was named *bunch1* and the classification of its corresponding phenotype was very easy and clear-cut, even as early as 50 days after sowing (Figure 1).

**Table 2:** **Segregation pattern of the spreading/bunch trait in several generations derived from closely related peanut varieties**.

**Generation**	**Hanoch (spreading) × Harari (bunch)**	**Harari (bunch) × Hanoch (spreading)**
F_1_	All spreading (5 plants)	All spreading (14 plants)
F_2_	238 spreading: 76 bunch	194 spreading: 58 bunch
F_3_ (families)	36 spreading: 81 segregating: 40 bunch	19 spreading: 37 segregating: 23 bunch

*In the F_3_ generation, each family contained 16 plants. Chi-square values of the 3:1 (F_2_) and 1:2:1 (F_3_ spreading:segregating:bunch, respectively) tests were all non-significant, indicating that data fit the expected segregation*.

### *Bunch1* is associated with several important agronomic traits

**Figure 2:**
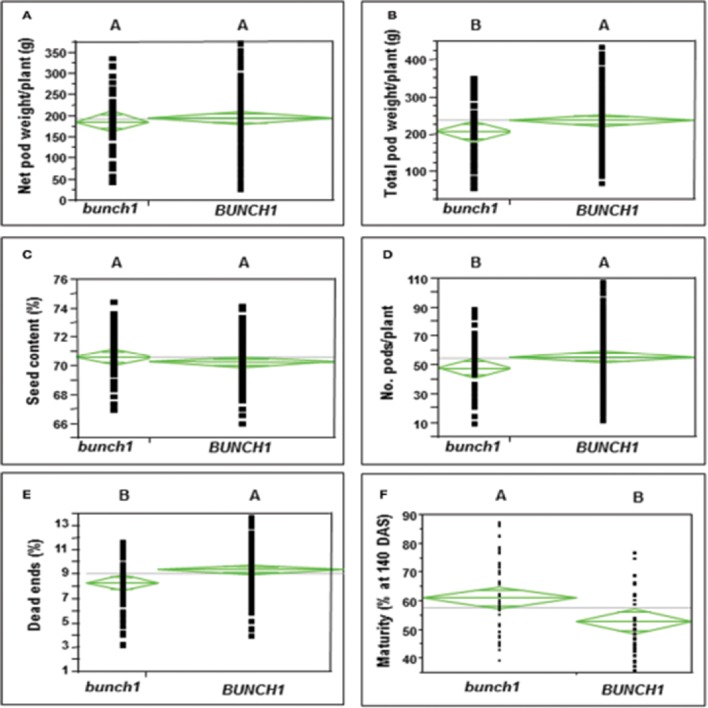
**The difference between the bunch (***bunch1***) and the spreading (***BUNCH1***) phenotypes of the ***bunch1*** gene in terms of several agronomic traits**. Phenotypes that are not labeled with the same letter are significantly different from one another (*p* < 0.05). **(A)** Net pod weight, **(B)** total pod weight, **(C)** seed content, **(D)** number of pods per plant, **(E)** dead-end ratio, and **(F)** percentage of mature pods at 10 days after sowing (DAS).

In addition to growth habit, other traits with agronomic importance were examined in the segregating populations. The associations between the *bunch1* phenotype and each of these traits are presented in Figure 2. The bunch phenotype of *bunch1* was significantly associated with a lower dead-end ratio. The *bunch1* phenotype was also found to have a small, but significant [Prob (t) = 0.0022] effect on early maturation. On the other hand, the *BUNCH1* phenotype (spreading) was significantly associated with higher total pod weight and a greater number of pods per plant.

### Identifying SNP markers that are linked to *bunch1*

In order to map *bunch1* on the peanut genome, a bulk segregant analysis was performed. For that analysis, 52 completely bunch and 47 completely spreading F_3_ families were bulked RNA was extracted from each bulk and converted into two libraries suitable for Illumina sequencing. After a cleaning procedure, 72 million reads per library (on average) were aligned to a 4X transcript assembly (peanutbase.org) that contains 120,364 peanut transcripts from both the A and B genomes [60,814 transcripts represent the A genome (*Arachis duranensis*) while 59,551 transcripts represent the B genome (*Arachis ipaensis*)]. With about 98% of reads mapped to the reference assembly, the expression levels of 117,957 peanut genes were measured.

**Figure 3:**
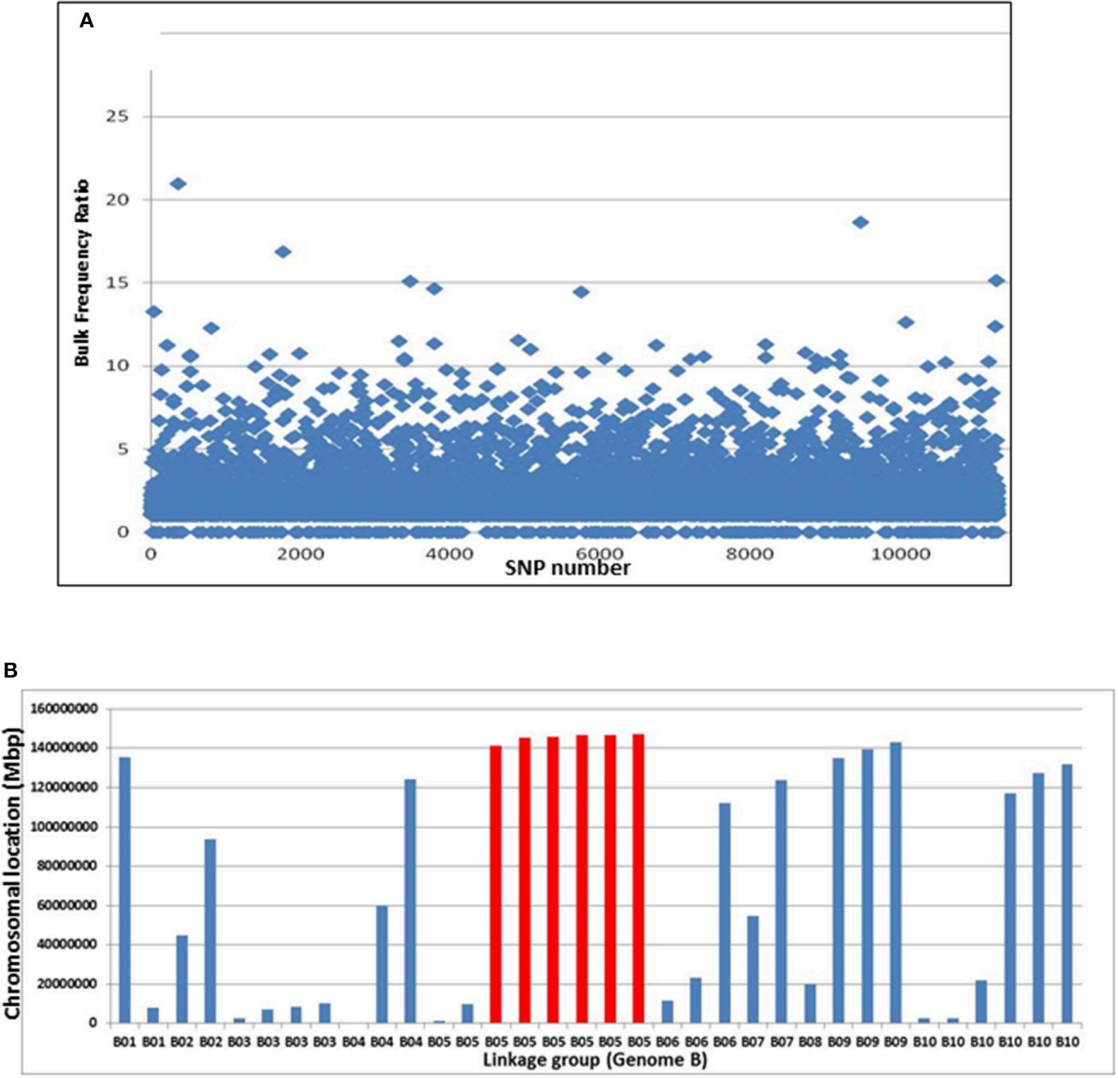
**Bulk frequency ratio (BFR) analysis to identify SNPs and genome locations that are linked to ***Bunch1***. (A)** The distribution of BFR in ~13,000 varietal-specific SNPs. **(B)** The genomic locations of the 34 SNPs with the highest BFR (>10), indicating one over-represented region at the end of linkage group 5B (red).

Pipelines for the SNP discovery and the analysis of bulk frequency ratio were constructed according to the general scheme that was previously suggested for polyploid wheat (Trick et al., 2012). After initial filtering, ~13,000 SNPs were found to be polymorphic by the two bulks. Subsequently, the bulk frequency ratio was determined for each SNP by calculating the frequency of each nucleotide of the SNP in each bulk and then dividing one bulk by another. If the SNP is a result of false-positive call of a homoeologus SNP, or if it is not linked to the trait, then both of the SNP nucleotides will be equally represented in the two bulks. However, if the SNP is linked to the gene, the frequencies of the SNP nucleotides in one bulk will be with significantly higher frequencies than the other bulk. In that manner, ~1,200 SNPs were found to have bulk frequency ratios of > 3, while 34 had bulk frequency ratios >10 (Figure 3A).

The genomic location of the 34 SNPs with bulk frequency ratios >10 within the peanut genome was recorded (Figure 3B, Supplemental Table 1). One region at the end of linkage group 5B was found to be over-represented in this SNP group; six of the 34 were located between 5B:135,963,343 and 5B147,304,662, including the SNP with the highest bulk frequency ratio [M875 (EZ721696.1); bulk frequency ratio = 23]. Two of these 5B linkage group and another few hypothetical SNPs with high BFR ratio from different linkage groups were further analyzed for SNP classification with Sanger sequencing. The purpose of this step was to roughly validate the location of *bunch1*. Therefore, samples from the parental lines and 6 F_2_ individual plants (from which 3 spreading and 3 bunch F_3_ families were derived) were selected (Supplemental Table 1). In this initial analysis, the SNPs from B5 linkage group were found almost perfectly segregating with trait, while the SNPs from the other genomic locations found to be either homoeologus SNPs (and not varietal) or didn't segregate with the trait (Supplemental Table 1), indicating for relatively high false positive ratio for the BSA by GBS technique in this system.

**Figure 4:**
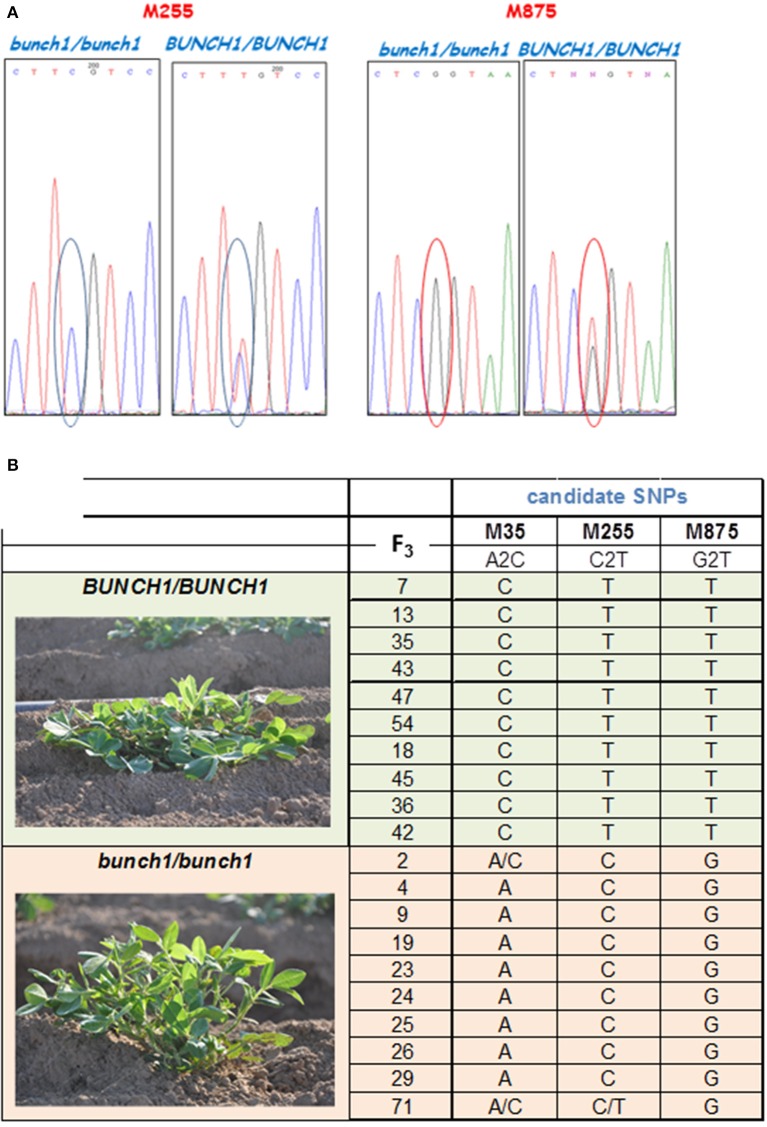
**SNP validation and linkage analysis with ***Bunch1***. (A)** An example of Sanger sequencing validation of two SNP markers. **(B)** Analysis of linkage between the top three SNP markers and the phenotype of *Bunch1*. Samples from 10 completely spreading (*BUNCH1*/*BUNCH1*) and 10 completely bunch (*Bunch1/Bunch1*) F_3_ families were analyzed.

SNP marker M875 and other two SNPs from the same genomic location that had high bulk frequency ratios [M35 (EZ721381.1), M255 (EZ748922.1)] were further analyzed (Figure 4A). Samples from 20 homozygous F_3_ families were checked (10 spreading and 10 bunch; Figure 4B). In this initial analysis, M875 was found to be completely linked to *bunch1*, while the two others, M35 and M255, were also linked to *bunch1*, but not completely. In the next phase, 182 F_4_ individuals that originated from heterozygous segregating F_3_ families were genotyped and phenotyped using markers M255 and M875. *Bunch1* was found to be located in a ~1.1 Mbp segment between markers M875 (B5:145,553,897; 1.9 cM) and M255 (B5:146,649,943; 2.25 cM).

### Further validation of the bulk segregant analysis using a peanut SNP array

**Figure 5:**
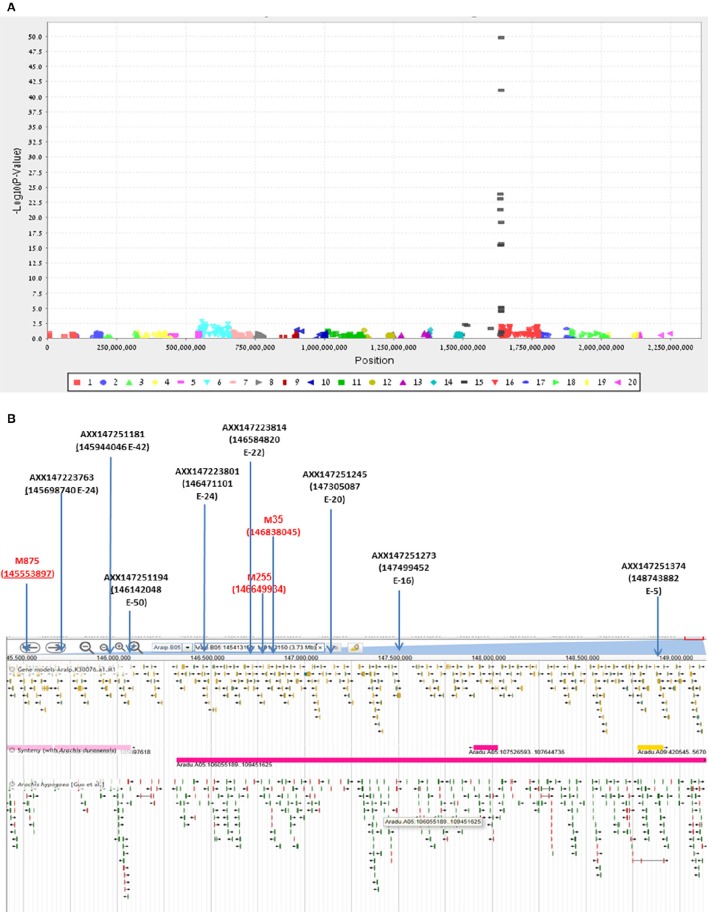
**Validation and further fine-mapping of ***Bunch1*** using an Affymetrix Axiom SNP array. (A)** Manhattan plot for the analysis of linkage between 615 Affymetrix Axiom SNPs and the *Bunch1* phenotype. 1–10 = genome A; 11–20 = genome B (e.g., 15 = linkage group 5B). **(B)** Integrative map for the bulk segregant analysis and SNP-array analyses of the peanut linkage group B5 (from PeanutBase.org). Markers derived from the bulk segregant analysis are indicated in red. Yellow–gene models. Green–ESTs of genes. Pink–syntheny of this region with *A. duranensis* (Genome A).

Final confirmation of the fine-mapping of the *Bunch1* gene was obtained using a new Affymetrix Axiom SNP array (Pandey et al., 2017). Since the chip technology is not efficient enough to distinguish between the heterozygous and homozygous states in the polyploid, a RIL population, which was advanced from the same cv. Hanoch × cv. Harari hybridization (F6:8), was used. Ninety-four RILs and the two parental lines were used for the analysis. Genomic DNA was extracted and applied to the 58,233 SNP clusters of the chip. Out of all of these SNPs, which were designed based on a wide spectrum of diploid and tetraploid peanut species, only 615 passed through the filtering pipeline, including significant differences between the parental lines and 1:1 segregation among the 94 RILs. The genetic analysis of these SNPs and the phenotype of *Bunch1* gene are presented in Figure 5A. Ten SNP markers from the array significantly (*p* < 0.01) co-segregated with the phenotype of *Bunch1* (Figure 5A). The best-linked SNP marker (AXX147251194) had only 1 recombinant RIL out of the 94 checked RILs (*p* = e^−50^; *R*^2^ = 0.92). All significant SNP markers were located in one region at linkage 5B, indicating once again that a single locus is controlling the branching habit trait in this background. These SNPs were located in very close proximity to the three SNPs that were derived from the bulk segregant analysis (Figure 5B). Interestingly, none of the SNPs from the bulk segregant analysis were detected in the chip array and vice versa, indicating that more SNPs could possibly be found by bulk segregant analysis and used in future SNP-array designs for cultivated peanut.

## Discussion

The genetic/molecular mechanism that controls growth angle in plants has been the subject of several studies, mainly involving monocotyledons, particularly rice. Several abnormal tiller-angle mutants and their corresponding genes have been reported in rice,) such as *LA1* (Li et al., 2007) and *PIN2* (Chen et al., 2012). Two additional genes with opposite effects on tiller angle, *Tiller Angle Control 1* (*TAC1*) and *Prostrate Growth 1* (*PROG1*), have also been identified in rice (Yu et al., 2007; Tan et al., 2008). These genes have played critical roles in the domestication of rice. There are several reports regarding the molecular biology of the growth angle of lateral shoots of dicot species. Roychoudhry et al. (2013) described a model in which the set point angle of lateral branches of higher plants is controlled by an auxin-dependent antigravitropic mechanism. The molecular basis for the spatial pattern of tree branches was also studied in peach, resulting in the discovery of a new ortholog of the *TAC1* gene, which controls the “pillar” tree phenotype (Dardick et al., 2013).

We explored the branching habit in the leguminous crop *Arachis hypogaea* and fine-mapped a major gene that controls this trait. The *bunch1* gene was mapped to a relatively small genomic region that includes ~70 ORFs for gene models. Interestingly, BlastX analysis showed that none of the above-mentioned genes that control the growth angle in either monocots or dicots were present in the peanut genome or mapped in approximation to *bunch1*. The genetic controller of *bunch1* may therefore be novel. Several candidate genes involved in plant hormone metabolism and light reception are located within that region and have been identified as possibly controlling *bunch1*. One of these may be a *FAR1-Related* sequence (B05:146200756.146203528) that encodes a family of proteins that are essential for phytochrome A-controlled far-red responses in Arabidopsis (*Arabidopsis thaliana*; (Lin and Wang, 2004). Another putative candidate gene is the 1-aminocyclopropane-1-carboxylate oxidase-like protein (ACC-oxidase; B05:146236653.146238358), which catalyzes the last step in ethylene biosynthesis. Ethylene biosynthesis may play an important role in determining peanut growth angle. Applying a relatively small amount of EPCA (an ethylene-releasing compound) caused the horizontal branches of runner-type plants to become erect (Ziv et al., 1976). Yet, these and other candidate genes must, of course, be further examined in light- and plant hormone-targeted studies, as well as subjected to verification by positional cloning and transformation.

The *Bunch1* gene had strong associations with several traits in the segregating F_2_ and F_3_ populations. Plants with the bunch phenotype had, on average, earlier maturity values and fewer dead-end pods. Plants with the spreading phenotype had on average more pods per plant, but many of those pods were actually undeveloped. In the bunch type, especially when a wide planting spacing is used (as in our experiments), many flowers are too far from the ground and cannot reach to the soil. For that manner, only the pods that are close to the root will develop. However, those pods that reach the soil develop in a more synchronized manner among the bunch types than among the spreading types. This promotes uniform maturation, better pod filling and, eventually, fewer dead-end pods. Indeed, many of the dead-end pods in the spreading types (like cv. Hanoch) are from distal parts of the branches, where pods develop late in the season and do not fully mature. In a subset of the RIL population, the bunch phenotype of the branching gene is significantly correlated with greater resistance to white mold (caused by *S. sclerotiorum*; data not shown; submitted for publication). Therefore, it is suggested that the phenotype of the *Bunch1* gene has an important agricultural role in Virginia-type peanuts.

As we explored the segregation patterns of several other crosses between Virginia-type related cultivars with different branching habits, we noticed that this model of the *bunch1* gene is relatively common within Israeli peanut breeding germplasm. Crosses between cv. Hanoch (spreading) and cv. Hillah/Shulamit (bunch) resulted in a 3:1 spreading:bunch ratio (data not shown), tracing the origin of this trait back to the early 1970s. Crosses between cv. Harari (bunch) and a runner-type peanut line GK-7-Ol (spreading) also resulted in a 3:1 ratio (data not shown), indicating that the single-gene model for this trait is not confined to the Virginia-type germplasm. Yet, in crosses with Valencia-type peanut germplasm (plants with an erect branching habit), this system of single-gene inheritance was not found andthe branching habit was therefore relatively hard to classify. We conclude that the *bunch1* phenotype is confined to the *A. hypogaea* ssp. *hypogaea* gene pool. However, other allelic variations of this gene may exist in the *A. hypogaea ssb. fastigiata* germplasm, since some of the bunch-habit Virginia-type lines in Israel (e.g., cvs. Shulamit, Hillah, etc.) have *A. fastigiata* origins. Moreover, the branching habit trait of peanut was analyzed in a previous study by Fonceka et al. (2012a), which involved mapping traits in a cross between amphidiploid *A. ipaensis* /*A. duranensis* and a Spanish-type cultivar (*A. hypogaea ssb*. fastigiata var vulgaris) with an erect growth habit. There was one significant QTL for the branching habit at the same location as *Bunch1* (at the end of linkage group B5), explaining 16.2 of the total variation of the trait in the population. It is very likely that the reported QTL for branching habit and the locus of *Bunch1* are the same. This demonstrates once again that the origin of the *Bunch1* phenotype may be beyond the *A. hypogaea* ssp. *hypogaea* genetic background.

We have demonstrated the relatively straight-forward and easy utilization of bulk segregant analysis for the fine-mapping of a monogenic trait in the low-polymorphic and heterozygous germplasm of cultivated peanut. Also, although the bunch/spreading trait is very easy to classify, there may be some new uses for these new markers in peanut breeding, particularly in the validation of successful F_1_ hybrids (when the female of the cross is the dominant spreading type) and the selection of homozygous spreading families in early breeding generations. The fine-mapping of this trait also provides a baseline for the cloning of *Bunch1*, presumably one of the first map-based positional cloning ventures in current genetic research of peanut.

## Author contributions

GK and YB were responsible for the molecular work; AF was responsible for the bioinformatic analysis; AP was responsible for RIL population analysis; IH conducted the field trials; RH managed the study and wrote the manuscript.

### Conflict of interest statement

The authors declare that the research was conducted in the absence of any commercial or financial relationships that could be construed as a potential conflict of interest.

## Supplementary material

Supplementary Table 1 **List of the 34 hypothetical SNPs with the highest (>10) BFR**. The table indicates the location of the SNP, the change of the nucleotide and the statistic score for the change. Also, the table presents further Sanger sequencing analysis of six SNPs with high BFR ratio. For this, six F_2_ individual plants were sampled, from which three spreading (green) and three bunch (yellow) F_3_ families were derived.
